# Sequencing-Based Measurable Residual Disease Testing in Acute Myeloid Leukemia

**DOI:** 10.3389/fcell.2020.00249

**Published:** 2020-05-08

**Authors:** Jennifer M. Yoest, Cara Lunn Shirai, Eric J. Duncavage

**Affiliations:** ^1^Department of Pathology, Case Western Reserve University, Cleveland, OH, United States; ^2^Department of Pathology and Immunology, Washington University in St. Louis, St. Louis, MO, United States

**Keywords:** measurable (minimal) residual disease, acute myeloid leukemia, next generation sequencing, unique molecular identifier, error-corrected sequencing, clinical applications of NGS, AML MRD

## Abstract

Next generation sequencing (NGS) methods have allowed for unprecedented genomic characterization of acute myeloid leukemia (AML) over the last several years. Further advances in NGS-based methods including error correction using unique molecular identifiers (UMIs) have more recently enabled the use of NGS-based measurable residual disease (MRD) detection. This review focuses on the use of NGS-based MRD detection in AML, including basic methodologies and clinical applications.

## Introduction

Acute myeloid leukemia (AML) is a heterogeneous group of clonal hematopoietic stem cell malignancies characterized by a block in myeloid differentiation resulting in increased myeloid blasts and rapidly aggressive clinical course (Estey and Dohner, 2006; O’Donnell et al., 2013; Papaemmanuil et al., 2016; Cai and Levine, 2019). With few exceptions for targeted treatments (e.g., midostaurin, enasidenib, etc.), risk-adapted therapy plays a major role in AML treatment decisions. Patients with low risk disease (e.g., core binding factor leukemias) are typically treated with standard induction and consolidation therapy whereas high risk patients (including those with complex karyotype or secondary AML) may be treated more aggressively, often with an allogeneic stem cell transplant after first remission (Schlenk et al., 2003; Dohner et al., 2017; Stein et al., 2017; Stone et al., 2017). However, the majority of AML patients remain intermediate risk at diagnosis, which makes risk-adapted treatment decisions less clear despite the widespread adoption of clinical sequencing-based panels that can identify recurrent gene mutations (Ivey et al., 2016). As large-scale sequencing studies of AML have shown, most AML patients harbor between 15 and 30 coding region mutations including driver and passenger mutations; the large number of combinatorial mutation profiles makes inferring individualized risk at diagnosis based on published studies difficult (Cancer Genome Atlas Research Network et al., 2013; Cai and Levine, 2019).

**FIGURE 1:**
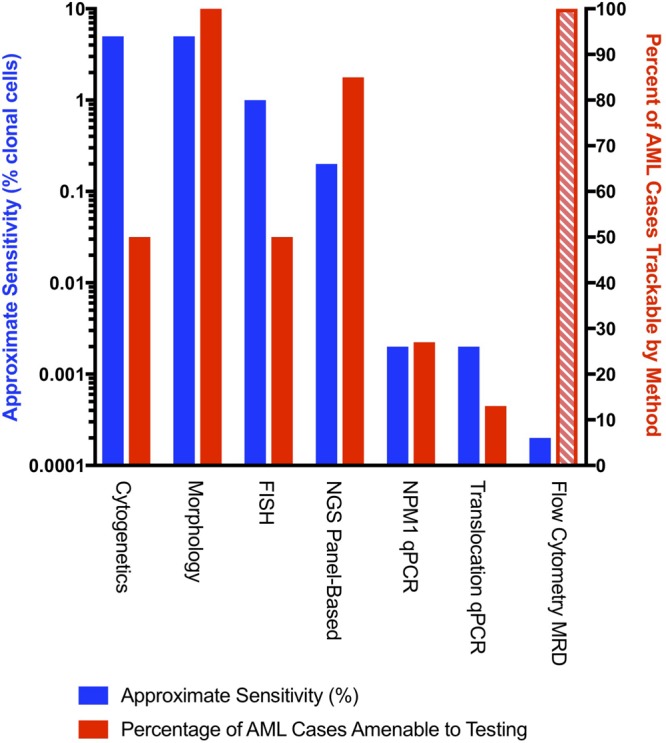
An approximate comparison of the sensitivity (left axis) and fraction of monitorable cases for commonly used AML MRD methods. Approximate sensitivity of molecular methods was converted from VAFs to number of mutated cells (double the VAF) to facilitate comparisons across methods. Cytogenetic analysis assumes that 50% of AML cases have at least one cytogenetic abnormality (Haferlach et al., 2012) with a detection sensitivity of one clonal cell in 20 metaphases. Morphologic evaluation assumes that a blast count of > 5% is considered relapse. FISH assumes a sensitivity of two clonal events in 200 evaluated metaphases. Panel-based NGS assumes a 40-50 gene panel capable of detecting at least one mutation in 85% of AML patients with a minimum sensitivity of 0.1% VAF (Cancer Genome Atlas Research Network et al., 2013). *NPM1* qPCR assumes a sensitivity of 0.001% VAF and an *NPM1* prevalence of 27% (Jongen-Lavrencic et al., 2018). qPCR for translocations assumes monitoring of inv(16), t(8;21), and t(15;17) with a prevalence of 13% (Papaemmanuil et al., 2016). Comparisons to AML MRD flow are complicated as the assay sensitivity depends to a large extent on the exact phenotype of the leukemia. The exact fraction of cases amenable to flow-based MRD is uncertain as indicated by the hashed line, but is modeled at 100% with a sensitivity of 0.0002% (Loken et al., 2012; Zhou and Wood, 2017; Schuurhuis et al., 2018). We also note that there may be discrepancies between MRD flow-based methods and molecular MRD (Jongen-Lavrencic et al., 2018).

Measurable residual disease (MRD; also referred to as minimal residual disease) testing in AML fulfills several unmet clinical needs, including the ability to determine individual risk in AML patients based on mutational clearance after treatment. MRD can be assessed by flow cytometry (Loken et al., 2012; Zhou and Wood, 2017) and a variety of molecular techniques (Yin et al., 2012; Hourigan et al., 2017; Vedula and Lindsley, 2017; Schuurhuis et al., 2018; Voso et al., 2019). Molecular MRD methods include single gene/fusion PCR-based monitoring (digital droplet and quantitative PCR) (Ivey et al., 2016; Mencia-Trinchant et al., 2017; Delsing Malmberg et al., 2019), chimerism-based studies (for post stem cell transplant patients) (Tsirigotis et al., 2016), TCR or IgH clonality-based MRD (possible in a subset of AML patients) (Zhong et al., 2018), and next generation sequencing (NGS)-based methods. A comparison of commonly used clinical MRD methods is summarized in Figure 1.

At AML diagnosis, mutation identification is important for determining patient risk and treatment. However, for the purpose of molecular-based MRD testing, the assumption is that clearance of disease-associated variants after therapy is associated with clinical outcome. While the idea of mutation clearance seems intuitive, there are several caveats including the persistence of “ancestral clones” which may represent return to a pre-leukemic age-related clonal hematopoiesis (ARCH) characterized by mutations in genes such as *DNMT3A*, *TET2*, and *ASXL1* (Jongen-Lavrencic et al., 2018); detection of these mutations may not represent persistent leukemia. Conversely clearance of other mutations thought to be acquired later in leukemogenesis, such as *FLT3* ITDs or mutations in activated cell signaling genes (*KRAS*, *NRAS*, etc.), may represent clearance of a subclone and not the leukemic founding clone (Bullinger et al., 2017). In essence, if any AML-specific mutation present at diagnosis is still detectable after treatment (e.g., 7 + 3, or allogeneic transplant), it follows that patients should be at higher *a priori* risk of relapse, regardless of the particular gene mutation. While conceptually simple, clearance-based molecular biomarkers in AML have been challenging to implement in the clinical setting for a variety of reasons related to both technical limitations and disease biology. One of the biggest technical challenges for AML MRD assays has been making them broadly applicable to most or all AML patients. Sensitive PCR-based AML MRD assays have been used for some time to monitor both recurrent translocations including core binding factor leukemias (Yin et al., 2012; Pigazzi et al., 2015; Ouyang et al., 2016; Schumacher et al., 2017) inv(16) and t(8;21) *RUNX1*-*RUNX1T1* and t(15;17) *PML-RARA* (Burnett et al., 2015; Brunetti et al., 2017) from RNA and can now be detected using multiplex RNAseq NGS-based approaches (Dillon et al., 2019). Specific recurrent gene mutations can also be monitored including *NPM1* (Salipante et al., 2014; Ivey et al., 2016) and *FLT3* (Bibault et al., 2015; Levis et al., 2018) from RNA or DNA. However, these methods are only applicable to a fraction of AML patients that carry these specific translocations or gene mutations. Further, some recurrently mutated single genes such as *FLT3* ITD are not always present at relapse or are unstable, thereby making them poor standalone targets for MRD (Shih et al., 2002; Garg et al., 2015). For this reason, DNA-based NGS panels that can detect multiple mutations in the majority of AML patients have become a popular approach to MRD detection. Consequently, this review will focus on DNA-based somatic mutation NGS monitoring methods for the detection of AML MRD in the clinical setting.

## Ngs Key Terms and Definitions

While translocation-driven leukemias (e.g., *PML-RARA*, *RUNX1-RUNX1T1*, and *CBFB-MYH11*) can be tested by qPCR with high sensitivity similar to AMLs with *NPM1* mutations, for most AML patients molecular-based MRD is not an option due to the lack of a robustly targetable mutation to measure. NGS-based MRD provides a way to monitor nearly all AML patients for molecular MRD by allowing for the detection of multiple gene mutations in a single assay. NGS-based MRD involves several key terms that will be explained in more detail including sensitivity [reported as variant allele frequency (VAF)], sequencing coverage, sequencing error rate, and assay breadth.

The VAF represents the fraction of reads containing a mutation divided by the total number of reads at a given locus and is a measure of mutational abundance. For example, a *U2AF1* p.S34F mutation present in 105 of 230 reads would yield a VAF of 45.6%. By clustering the VAFs from different mutations, one can reconstruct the clonal architecture of the leukemia (Ding et al., 2012; Welch et al., 2012); mutations with VAFs similar to the *U2AF1* mutation in this case (∼45%) are presumably contained in the same clone. The clone with the highest mutation VAF (after adjusting for copy number changes) is presumed to be the “founding clone,” while mutations with lower VAFs represent distinct subclones that contain the founding clone mutation (in this case *U2AF1*) plus the lower VAF mutation(s). The maximum sensitivity for NGS-based assays is also defined in terms of VAF. For example, an assay with a maximum sensitivity of 1% VAF would correspond to one mutant cell (with heterozygous mutations) in 50 normal cells. Similarly, an assay with a maximum detection sensitivity of 0.1% would detect one mutant cell in 500 normal cells.

**FIGURE 2:**
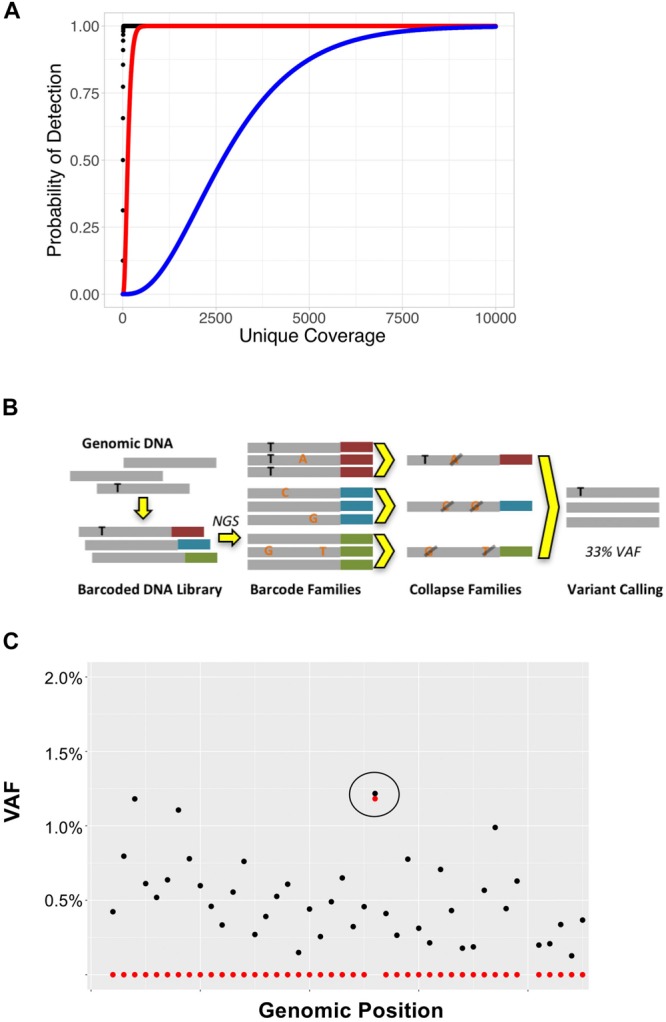
**(A)** Coverage depth required to detect variants at various VAFs. Binomial sampling probability for detection of variants with VAFs of 50% (black), 2% (red), and 0.1% (blue) assuming each variant must be seen at least twice. **(B)** Overview of UMI-based sequencing. Fragments of genomic DNA are first ligated to unique barcode sequences (red, blue, and green). Libraries are then sequenced to high coverage depths, such that each DNA molecule is sequenced at least 3x. Read families with the same barcode are collapsed such that only variants present in > 90% of reads (black) are retained; variants present in only a subset of reads (orange), representing sequencing error, are discarded. **(C)** UMI-based error correction using cell line DNA with *BRAF* p.V600E mutation. In this example, cell line DNA was diluted such that the BRAF p.V600E was 1.3%. DNA was then sequenced to 10,000x total coverage. The plot represents the *BRAF* genomic locus containing the p. V600E mutation (circled). Black points represent VAFs of variants detected without error correction and red points represent the VAFs of variants detected using UMI-based error correction on the same data. While the raw data (black) show background sequencing errors making it difficult to discern the true positive variant from noise at this locus, application of error correction (red) reduces background noise leaving only the true positive variant.

In NGS-based assays, coverage is defined as the number sequencing reads that span (or cover) a particular locus. Coverage can be expressed in terms of “total coverage” or “unique coverage,” although not all sequencing methods are able to differentiate the two. Unique coverage refers to the number of reads that come from distinct DNA molecules and can be used to infer the maximal sensitivity of an NGS assay. For example, if a locus has 100x unique coverage and 10,000 total coverage, the maximum sensitivity for this locus is 1/100 = 1% VAF, as there were only 100 unique DNA molecules sequenced (Figure 2A). The duplicate rate in this example is 10,000/100 = 100x; duplicate reads descend from the same DNA molecules, and there is no new information contained in these reads. While standard NGS analysis pipelines will generally discard duplicate reads, they can be useful for sequencing error correction as described below. In order to achieve high detection sensitivities for MRD detection, NGS assays must have high numbers of unique reads. This can be obtained by using large DNA input amounts, sequencing to high total coverage depths (to ensure all DNA molecules are sequenced), and using efficient enrichment methods that allow for a high fraction of input DNA molecules to be sequenced. It is also important to note that not all sequencing enrichment technologies allow for differentiation between duplicate and total reads. For this reason, pure PCR-based enrichment methods are not ideal for MRD, unless steps can be implemented to distinguish duplicate reads originating from the same DNA templates from unique reads which originate from different template molecules.

Like all measurements, base calling by NGS is subject to error. This error rate is partly due to the intrinsic properties of the sequencing platform (e.g., Illumina and Thermo Fisher) as well as errors that occur during PCR amplification (Schmitt et al., 2012). While the sensitivity of an NGS assay can be increased by increasing unique coverage, the specificity cannot be increased with increased coverage depths; therefore, calling variants below the intrinsic error rate will result in large numbers of false positive calls. For most standard clinical NGS pipelines, the limit of detection is 2–5% VAF, well above the ∼1% error rate associated with most sequencers (Spencer et al., 2014; Salk et al., 2018). However, the desired sensitivity for MRD applications is generally 0.01–0.5% VAF, well below the error rate of the sequencing process. There are several approaches to overcoming problems with the error rate, including computational methods and physical error correction methods as described below.

## Error Corrected Sequencing Methods

A key component necessary for NGS-based AML MRD assays is high sensitivity, or sequencing below the error rate of the sequencing platform, which requires additional steps to improve the error rate and reduce false positive calls. There are many computational approaches to reducing sequencing error rates that do not require physical changes to sample library preparation (Prosperi and Salemi, 2012; Gerstung et al., 2014; Lee et al., 2017; Zhao et al., 2017). The simplest computational approaches rely on removing reads with high base error rates (Q scores < 30) and low mapping scores (Spencer et al., 2014). More complex methods use background error rate models that are generated by sequencing numerous control specimens to determine the baseline error rate for each position in the targeted region. For example, comparing base counts for a single position in 100 normal cases where the expected reference base is a C may indicate that 0.2% of calls are A, 0.1% of calls are G, and 0.1% of calls are T. If an MRD sample has a T variant detected at this position with a VAF of 0.4%, various statistical measures may be used to determine whether this variant represents background error or a true positive event. While fairly simple to implement, these methods are highly susceptible to batch effects including factors that may affect the error rate such as instrument cluster density, PCR conditions, and sequencer variability. Improved error rate modeling can be obtained using in-run controls; however, these methods can be cumbersome and generally have limited performance improvements in the clinical laboratory (Vallania et al., 2010; Spencer et al., 2014).

Another important factor that may alter clinical error rates in NGS-based MRD assays is prior knowledge of mutations present at diagnosis. For example, the positive predictive value of a patient with a specific *TP53* mutation at diagnosis and subsequent detection at low VAF post treatment is higher than a patient with no prior sequencing data and the same mutation detected post treatment. In the former, the prior probability of the low VAF post-treatment mutation being a true positive is high whereas in the latter example, in which low VAF mutations are essentially being discovered *de novo*, the mutation may represent a false positive depending on the specificity of the assay. It should also be noted that the error rates for other classes of mutations such as insertions/deletions differ from that of single nucleotide variants and is in general much lower. This observation can be leveraged in MRD assays that target common recurrent indels such as *FLT3* ITDs or *NPM1* insertions (Salipante et al., 2014; Lin et al., 2015; Zhou et al., 2020).

Physical error correction methods generally involve changes to library preparation to incorporate unique molecular identifiers (UMIs) in sample DNA (Salk et al., 2018). UMIs are DNA oligonucleotides that are added to genomic DNA before amplification or capture and allow for tracking of individual DNA molecules throughout the sequencing process (Kinde et al., 2011). UMI-based error corrected sequencing has found numerous applications in addition to detection of MRD including detection of subclonal hematopoiesis and rare pre-existing mutations (Wong et al., 2015; Young et al., 2016; Duncavage et al., 2018). UMIs can range in length from 3 to 16 nucleotides and may be random sequences (synthesized as degenerate oligonucleotides) or have fixed sequences. In general, UMIs are ligated to sample DNA during the first steps of the library preparation process to avoid problems with early cycle PCR errors. Once a sample is sequenced, UMIs allow for sequenced reads to be tracked back to individual input DNA molecules (Figure 2B). UMI-based error correction leverages duplicate reads to determine whether a variant is an error or a true mutation by comparing all the reads that descend from a single DNA molecule (i.e., share the same UMI) to one another. Duplicate reads that contain the same UMI are referred to as a “read family.” During the error correction process, reads that belong to the same read family are collapsed such that only variants present in all (or most) members of the read family are included in the final consensus read. Using this approach, random sequencing errors can be corrected, as a true positive variant should be present in all of the reads generated from a single UMI-tagged DNA molecule (Figure 2C); errors or false positive calls will be present in only a subset of read family members.

Currently, there are various vendor and laboratory-derived approaches for UMI-based sequencing including Agilent Haloplex HS, Agilent SureSelect XT HS, IDT xGen Dual Index UMI, New England Biolabs NEBNext Direct, smMIPS (Hiatt et al., 2013), ArcherDx, and modifications of other commercial kits (Wong et al., 2018). While all approaches are similar in concept, they can be further divided by single UMI and duplex UMI-based error correction. Duplex UMI methods differ from single UMI methods in that both DNA strands are tagged with complementary UMIs making it possible to determine whether an observed mutation was initially present in both DNA strands and therefore less likely to be an artifact. In the ideal setting, single UMI error correction can achieve an error rate of 3.4 × 10^–4^, while duplex-based methods are capable of reducing the error rate by at least two orders of magnitude to 2.5 × 10^–6^ (Schmitt et al., 2012). Performance in the clinical laboratory when sequencing a panel of genes is generally much lower and often limited to VAFs of 0.1–0.5% (Duncavage et al., 2018; Kim et al., 2018). Despite the potential increase in sensitivity and specificity achieved with duplex error correction, most clinical AML MRD methods rely on single strand UMI-based error correction, since the coverage required to realize performance gains with duplex UMI error correction across a panel of genes is generally not tenable in the clinical laboratory.

## Design Challenges for Clinical NGS-Based AML MRD

Next generation sequencing-based MRD can be implemented in many ways in the clinical laboratory, and at present the optimal solution to NGS-based tracking is unclear. Key design challenges revolve around the compromises between breadth vs depth of an MRD assay and one-size-fits-most assays vs patient-specific, bespoke assays.

Acute myeloid leukemia is composed of a founding clone and one or more subclones that contain the founding clone mutations plus additional mutations (Welch et al., 2012). Most AMLs will harbor between 15 and 30 somatic coding region mutations. A subset of these mutations occur in a core group of frequently mutated genes such as *FLT3*, *NPM1*, *DNMT3A*, etc.; however, the remainder of mutations are non-recurrent and require broad sequencing approaches to identify (Cancer Genome Atlas Research Network et al., 2013). Therefore, tracking all mutations (and therefore all clones) present in an individual AML patient requires a “wide” approach and an assay with sufficient breadth such as exome or whole genome sequencing. While a “wide” approach will permit monitoring of all clonal mutations in nearly all AML patients, it will be limited by a low depth of coverage in a wide target space; high depth of coverage is needed for a highly sensitive MRD assessment, especially compared to other MRD techniques such as flow cytometry and single-gene PCR-based assays. For example, most exome sequencing is performed at coverage depths of 100–500x, permitting detection of variants in the range of only 10–2.5% VAF (Tyner et al., 2018).

“Deep” approaches to NGS-based MRD have the advantage of increased sensitivity due to high coverage depths (often > 20,000x) but may lack the ability to track all clonal mutations. “Deep” approaches can be implemented in two ways: using a patient-specific approach or a fixed panel of genes. In the patient-specific approach, somatic mutations are first identified using exome or whole genome sequencing of “diseased” bone marrow and “normal” skin (or other source of normal DNA) (Tyner et al., 2018). Probes are then constructed to detect these mutations in subsequent samples using high-coverage sequencing, allowing for high detection sensitivities for all mutations detected in the diagnostic sample. The fixed panel of genes approach relies on the observation that nearly all AML cases have mutations in a small core group of genes (presumably involved in disease pathogenesis) that can easily be sequenced to high coverage depths. Since fixed panels do not cover all mutations present in a patient, they may not be sufficient to track all clones present in a patient. In addition, both “deep” approaches will also miss “rising clones” or newly acquired mutations that are often associated with AML relapse (Wong et al., 2016).

Generating NGS-based MRD data in the CLIA-regulated clinical environment in a clinically relevant time-frame presents additional challenges; therefore, most studies of NGS-based MRD to date have been retrospective studies. While patient-specific deep sequencing approaches as described above are capable of highly sensitive monitoring of all mutations in an AML patient, they are logistically challenging to implement in the clinical setting. The generation and analysis of initial paired tumor/normal exome or whole genome data may take many weeks. Design, manufacture, and validation of targeted probes based on mutations detected in the diagnostic sample take additional time, making these methods impractical for MRD monitoring based on standard post treatment time points (e.g., day 14 or day 30). Deep panel-based NGS is far easier to implement in the clinical setting and can be used with standard post treatment time points.

In addition to design challenges associated with clinical NGS-based MRD, there are also interpretive challenges. A major potential pitfall of NGS panel-based MRD interpretation is the detection of persistent age-related clonal hematopoiesis (ARCH), which may not represent AML (Genovese et al., 2014; Jaiswal et al., 2014; Steensma et al., 2015). For example, in a study of NGS-based AML MRD, Jongen-Lavrencic et al. (2018) noted the persistence of so-called “DTA mutations” in *DNMT3A*, *TET2*, and *ASXL1* after chemotherapy which were not correlated with an increased relapse rate. This finding was also observed in post allogeneic transplant patients by Thol et al. (2018). In the clinical setting, unless an AML patient had persistence of other non-DTA mutations, it would be difficult to interpret persistent DTA mutations as persistent AML. Another potential interpretative issue is that most clinical AML NGS assays rely on “tumor only” sequencing and do not use paired normal tissue to assure somatic status of a mutation. It is therefore possible that constitutional variants incorrectly assigned somatic status could be interpreted as persistent molecular disease, especially if VAFs of these variants are skewed from the normal heterozygous VAF for technical reasons.

Likely the single biggest challenge to widespread clinical implementation of NGS-based MRD is the lack of payer coverage for such testing in the United States. While flow cytometry-based MRD evaluation is generally covered by payers under existing CPT codes for most standard surveillance time points, panel-based NGS testing is generally covered only at initial diagnosis and relapse, but not for MRD testing purposes when the patient is in complete morphologic remission. For NGS-based MRD testing to have an impact in the clinical setting, local and national coverage determinations will have to be amended to include NGS testing at surveillance time points. Further, under existing CPT codes, current reimbursement rates are often too low to cover the costs of high-coverage sequencing-based MRD evaluation. Due to these practical challenges, most clinical NGS-based MRD testing in AML is confined to clinical trials at present.

## Clinical Studies of NGS-Based MRD

In one of the earliest examples to show the clinical relevance of NGS-based MRD, Klco et al. (2015) used exome or whole genome sequencing in 50 diagnostic AML samples and paired normal tissue to first identify somatic mutations and then used either exome or targeted sequencing of bone marrow biopsies taken 30 days after chemotherapy to determine whether mutations had cleared. Even using a fairly insensitive approach (VAF cut-off of 2.5%), they found that patients who cleared their somatic mutations had longer event-free and overall survival with hazard ratios of 6.0 (CI 1.93–7.11) and 2.86 (CI 1.39–5.88), respectively.

Jongen-Lavrencic et al. (2018) used a 54 gene panel on 482 AML patients who achieved a complete remission of which 430 (89.2%) had at least one detectable mutation prior to standard induction chemotherapy. They then sequenced bone marrow DNA post treatment using computational error correction to achieve a maximum sensitivity of 0.02% for previously identified mutations and found that persistent molecular disease was correlated with significant decreases in relapse-free survival (hazard ratio of 1.92; CI 1.46–2.54) and overall survival (hazard ratio of 2.06; CI 1.57–2.91) after excluding “DTA mutations” as described above. Similar observations were seen in multivariate analysis. In addition, the group compared NGS-based MRD to flow cytometry-based MRD. Specifically, they were able to demonstrate that agreement between the two methodologies in either direction strengthened the resulting correlations with outcome, but disagreement between the two methodologies in either direction (flow positive/NGS negative or vice versa) defined a group of patients with similar intermediate prognosis. Importantly, they concluded that NGS-based molecular MRD had a significant additive prognostic value when combined with flow cytometric MRD studies.

In a similar study, Morita et al. (2018) evaluated 131 AML patients who achieved complete remission of which 122 (93%) had at least one mutation detectable with a 295 gene panel. Thirty day post-induction bone marrows were then sequenced using maximum VAF cut-offs of 2.5%, 1.0%, and undetectable. The authors found that day 30 VAFs < 1% were associated with significantly better overall survival than that of patients with detectable mutations > 1% VAF. Patients who had undetectable mutations at day 30 had significantly better event-free survival in multivariable analysis after adjusting for age, cytogenetic risk, allogeneic stem-cell transplantation, and flow cytometry-based minimal residual disease. Removal of DTA mutations from the analysis resulted in stronger prognostic associations.

Next generation sequencing-based MRD has also been used in AML allogeneic transplant setting. Thol et al. (2018) used a 46 gene sequencing panel to identify mutations in 116 pre-transplant AML patients, identifying at least one trackable mutation in 93% of patients. High-coverage UMI-based error correction of pre-allogeneic transplant blood or bone marrow specimens with a sensitivity of < 0.02% demonstrated that 45% of patients were MRD positive with a median VAF of 0.33% (range 0.016–4.91%). These pre-transplant MRD positive patients had a higher cumulative incidence of relapse according to a competing risk analysis (hazard ratio 5.58; *P* < 0.001). No difference, however, was observed in overall survival between MRD positive and negative patients. Similarly, Press et al. (2019) used a 42 gene NGS panel with a sensitivity of 0.5% VAF to evaluate 42 AML patients for MRD before and after allogeneic transplant. They found that the cumulative incidence of relapse was significantly higher in pre-transplant MRD positive patients (*P* = 0.014). In multivariate analysis, pre-transplant MRD positivity was associated with a higher relapse risk (hazard ratio = 7.3; *P* = 0.05), shortened progression free survival (*P* = 0.038), and marginally shortened overall survival (*P* = 0.068). A third study by Hourigan and colleagues (Bloomfield et al., 2018) tested pre-transplant blood samples from AML patients in morphologic CR who were randomly assigned to either myeloablative or reduced-intensity pre-transplant conditioning using a 13 gene NGS panel. For patients with a detectable mutation, they found significant differences in relapse (19 vs 67%; *P* < 0.001) and overall survival (61 vs 43%; *P* = 0.02) between patients with myeloablative or reduced-intensity pre-transplant conditioning, respectively. In multivariate analysis, they found pre-transplant NGS positive patients who underwent reduced-intensity pre-transplant conditioning had a higher risk of relapse (hazard ratio 6.38; 95% CI 3.37 to 2.10), decreased relapse free survival (hazard ratio 2.94; 95% CI, 1.84–4.69), and decreased overall survival (hazard ratio 1.97; 95% CI, 1.17–3.30) compared to patients who underwent myeloablative pre-transplant conditioning. These data suggest that myeloablative conditioning may improve outcomes for MRD positive pre-transplant patients.

Kim et al. (2018) reported similar findings using a cohort of 104 AML patients who underwent allogeneic stem cell transplant. Using a panel of 84 genes on pre-transplant bone marrows and paired CD3 + T-cells, 90 patients (81%) of the 104 had a trackable mutation. Post-transplant bone marrow samples were collected 21 days after transplant and sequenced using computational error correction with a VAF sensitivity of 0.2%. The authors found that post-transplant MRD positive status was associated with an increased risk of relapse (56.2 vs 16.0% at 3 years; *P* < 0.001) and reduced overall survival (36.5 vs 67.0% at 3 years; *P* = 0.006) compared to MRD negative patients. These associations remained significant in multivariate analysis when taking in to account European LeukemiaNet risk groups.

In another recent publication, Balagopal et al. (2019) used a UMI error corrected hybrid capture enrichment panel targeting 22 recurrently mutated genes and tested 30 post-transplant AML patients who were negative for MRD by conventional short tandem repeat (STR)-based testing. STR testing is frequently used to monitor post bone marrow transplant patients in the clinical setting and works by comparing differences in highly polymorphic alleles between recipient and donor bone marrow via PCR and capillary-based fragment sizing. If a transplant patient is fully engrafted, the marrow will show an STR pattern consistent with the donor with no evidence of the recipient’s DNA. The sensitivity for STR based studies is approximately 2.5%. In their study, Balagopal et al. found that 18 of 30 (60%) of post-transplant AML patients who were negative by STRs had a detectable clone by their NGS assay. They also found a high concordance between blood and bone marrow samples, suggesting that blood could be a surrogate for marrow.

Clinical studies are summarized in Table 1.

**TABLE 1:** Summary of NGS-based multi-gene MRD studies in AML.

					**Study**	**Mean**	**Maximum**
					**size**	**MRD**	**sensitivity**
**Author**	**Year**	**Key findings**	**Disease state**	**Technique**	**(*n*)**	**coverage**	**(VAF)**
Klco	2015	Clearance of disease-specific mutations at 30 days post induction correlated with better EFS and OS	AML, post induction	WES or WGS, and amplicon-based sequencing with paired normal tissue	50	543X (exome) 14,780X (amplicon)	2.5%
Jongen- Lavrencic	2018	Persistence of non-“DTA” mutations correlated with decreased RFS and OS. Combining NGS and flow MRD data produced strong correlations with outcome when methods were concordant and defined an intermediate prognosis group when methods were discordant	AML, post induction	54 Gene tumor-only NGS panel with position-based error correction	482	Not stated	0.02%
Morita	2018	Patients with clearance of disease-associated mutations (<1% VAF) at 30 days post induction had better OS and better EFS after multivariate analysis; this correlation was strengthened by the exclusion of “DTA mutations” from the analysis	AML, post induction	295 Gene tumor only NGS panel	131	575X	<1.0%
Thol	2018	Detection of disease-associated mutations post-transplant was associated with higher incidence of relapse, but no difference in OS	AML, pre- transplant	46 Gene custom amplicon panel with UMI-based error correction	116	6,100x	0.02%
Kim	2018	MRD positive patients had a higher incidence of relapse and lower OS	AML, post-transplant	84 Gene NGS panel with paired normal T-cells and position-based error correction	104	1726X	0.02%
Balagopal	2019	Found evidence of MRD in 18/30 (60%) of post-transplant AML patients who showed no evidence of disease by standard engraftment studies	AML, post-transplant	22 Gene panel with UMI-based error correction	30	>10,000X	0.1%
Press	2019	NGS MRD positive pre-transplant patients had higher risk of relapse in multivariate analysis	AML, pre- transplant	42 Gene panel, coverage-based error correction	42	1900x	0.5%
Hourigan	2019	NGS MRD positive pre-transplant patients with reduced-intensity conditioning had increased relapse rates, decreased overall survival, and decreased OS compared to patients who underwent myeloablative conditioning	AML, pre- transplant	13 Gene panel with UMI-error correction run in peripheral blood samples.	190	>100,000x	0.001%

*Abbreviations: EFS, event free survival; OS, overall survival; NGS, next generation sequencing; WES, whole exome sequencing; WGS, whole genome sequencing; RFS, relapse free survival; MRD, measurable residual disease; UMI, unique molecular identifier.*

Another promising avenue for NGS-based MRD testing is “liquid biopsies” or cell-free DNA assays which use many of the same techniques as previously described for detecting MRD in bone marrow samples and have recently been investigated as a potential methodology for AML MRD analysis by Nakamura et al. (2019). These circulating tumor DNA (ctDNA) methods are more commonly used for solid tumors including in the clinical setting (Merker et al., 2018). Although their study demonstrated proof of concept by detecting mutations down to a VAF of 0.04% from a blood sample and would spare patients the discomfort of repeated bone marrow biopsies, the digital droplet PCR (ddPCR)-based methodology is likely too cumbersome for use in the clinical setting as it requires patient-specific primer design and validation. NGS panel-based ctDNA MRD methods, however, that have been successfully used for solid tumors and lymphoma could be applied to AML (Newman et al., 2014; Kurtz et al., 2018). Future studies will have to determine whether ctDNA or peripheral blood leukocytes are a more sensitive substrate for AML MRD.

## Considerations and Future Directions

Next generation sequencing-based MRD evaluation of post-transplant AML patients has been shown to predict clinical outcome. A major advantage of NGS-based monitoring methods is that they are applicable to nearly all AML patients, unlike translocation-based or single gene molecular MRD assays that are only available to the minority of AML patients with recurrent translocations or NPM1 mutations. At present, there are limited data comparing NGS-based MRD testing to flow cytometry based MRD in AML; however, one of the major potential advantages of NGS-based MRD is that it is less subjective than flow cytometry-based approaches, and may therefore be a more reliable measure of MRD status. Another potential advantage of NGS-based MRD methods over flow cytometry-based MRD is that they appear to show similar results in blood and bone marrow as they detect mutations present in mature cells and do not rely on phenotypic aberrancies present only in rare bone marrow blasts (Duncavage et al., 2017). Potential disadvantages of NGS-based MRD over flow cytometry-based MRD include longer turnaround times, increased assay costs, and uncertain payer reimbursement. Since the detection targets are distinct, NGS MRD and flow cytometry-based MRD approaches may complement one another, as suggested by Jongen-Lavrencic et al. (2018). Additional studies are needed to better define the optimal clinical use for NGS and flow-based MRD methods.

In addition to predicting clinical outcomes, NGS-based MRD assays can be used to monitor treatment response in AML patients (Uy et al., 2017). Such assays could potentially be used to define more objective surrogate “molecular clearance” endpoints in clinical trials. They could also be used to define early treatment response, determine therapeutic efficacy of new drugs or drug combinations, or to understand the clonal/mutational dynamics caused by specific therapies. Since NGS-based assays can be run from blood and do not necessarily require a bone marrow biopsy, more frequent monitoring of patients could be performed during clinical trials.

While multiple studies have demonstrated the utility of NGS-based MRD assays to predict clinical outcome, it remains unclear whether therapeutic intervention based on these data will ultimately change patient outcomes and result in increased overall survival (Hourigan et al., 2019). It also remains unclear exactly what level of mutation clearance is required (i.e., VAF) to determine whether a patient should be deemed MRD negative and how issues surrounding persistent clonal hematopoesis should be resolved. Future clinical trials will be required to evaluate the effects of early therapeutic intervention based on MRD positive post-treatment findings.

## Author Contributions

JY and ED conceptualized the manuscript and its organization. JY, CS, and ED all participated in writing the manuscript.

## Conflict of Interest

The authors declare that the research was conducted in the absence of any commercial or financial relationships that could be construed as a potential conflict of interest.
